# Living in the tide of change: explaining Japanese subjective health from the socio-demographic change

**DOI:** 10.3389/fpsyg.2014.01221

**Published:** 2014-10-29

**Authors:** Hidefumi Hitokoto, Junko Tanaka-Matsumi

**Affiliations:** Department of Psychological Sciences, Kwansei Gakuin UniversityNishinomiya city, Hyogo, Japan

**Keywords:** socio-demographic change, cultural happiness, individualism-collectivism, health, regional culture

## Abstract

Today, countries around the world are caught in the tide of change toward *Gesellshaft*, or individualistic socio-demographic condition. Recent investigations in Japan have suggested negative impacts of change on emotional and motivational aspects of the Japanese self (Norasakkunkit et al., 2012; Ogihara and Uchida, 2014). Building on previous findings, in Study 1, we measured socio-demographic change toward individualistic societal condition during 1990–2010—two decades marked by great economic recession—at the levels of prefecture and city using archival data. In Study 2, we tested whether Japanese adults' general health, satisfaction with life, self-esteem, and perceived social support were negatively predicted by the change using social survey. Results of hierarchical linear modeling showed small but unique negative effects of the change on several health measures, suggesting that this change had an impact on health, above and beyond individual personality traits, and demographics. Additionally, interdependent happiness, the type of cultural happiness grounded in interdependence of the self (Hitokoto and Uchida, 2014), showed an independent positive relationship with all aspects of health examined. Implications for health studies in changing socio-demographic condition are discussed in the context of Japanese society after economic crisis.

## Introduction

Studies of culture change have paid attention to the change in the *level* of national values (Hofstede, 2001; Allen et al., 2007; Bilsky et al., 2011; Hamamura, 2012; Twenge et al., 2012), but few studies have been conducted on the *relationship* between the change and health of residents (Oishi et al., 2011). We argue that change in the socio-demographic conditions would undermine individuals' psychological health, because whereas human well-being is a function of habituated behavioral tendencies fitted best to accustomed context, change will bring alterations to the requirements of the external environment. We believe such process is apparent across national boarders. In this study, we focus on the typical case of socio-demographic change which took place in Japan.

After the economic crisis known as the collapse of the *bubble economy*,^1^ Japan has witnessed increasing societal problems, and this has led to investigations on the relationship between culture change and health (Norasakkunkit et al., 2012). In this study, we take the perspective that Japanese culture, especially its socio-demographic context is becoming more non-traditional (Hamamura, 2012), and the change in context from 1990 to 2010—the two decades of extremely low rate of new employment opportunities, spread of new liberalism, and market globalization (Zielenziger, 2006)—would negatively explain the health of Japanese adults.

### The worldwide tide of change

**Figure 1:**
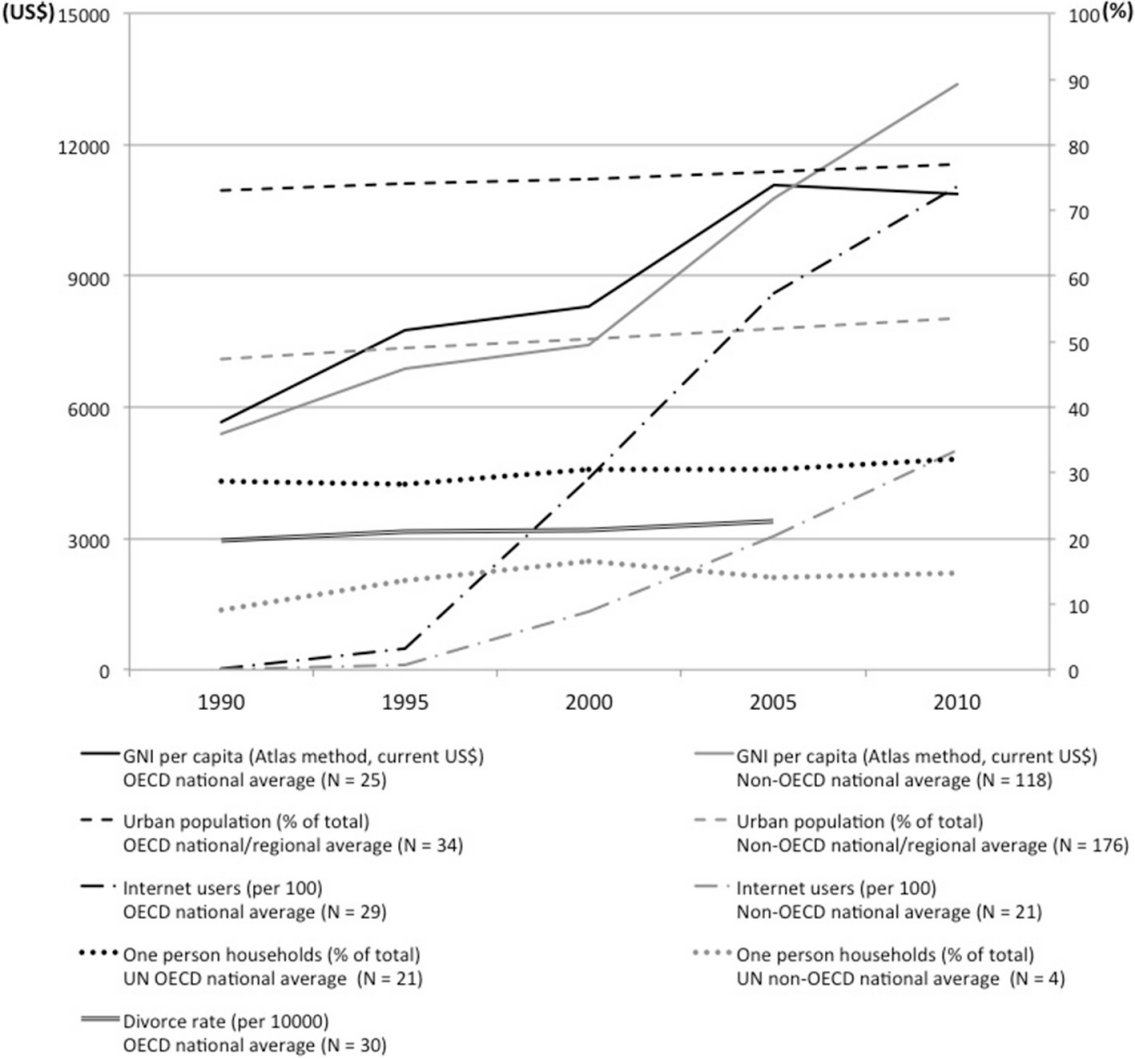
**Socio-demographic change toward *Gesellshaft* around the world during 1990–2010 for OECD and non-OECD nations**. OECD nations are United Kingdom, Germany, France, Italy, Netherlands, Belgium, Luxembourg, Finland, Sweden, Austria, Denmark, Spain, Portugal, Greece, Ireland, Czech Republic, Hungary, Poland, Slovak Republic, Estonia, Slovenia, Japan, United States, Canada, Mexico, Australia, New Zealand, Switzerland, Norway, Iceland, Turkey, Korea, Rep., Chile, and Israel.

The world shift toward *Gesellshaft* (Tönnies, 1988) or socio-demographic condition common in individualistic societies (Greenfield, 2013) is one of the largest contextual changes surrounding recent human ecology. Starting from economically developed countries, urban population, internet users, one person households, divorce, and national affluence are increasing throughout the world. Conceptually, we regard these changes as indicating the change toward increased *Gesellshaft* socio-demographic condition. Figure 1 shows a world summary of the changes, summarized from the database of OECD (Organization for Economic Cooperation and Development: http://www.oecd-ilibrary.org), United Nations Economic Commission for Europe (http://w3.unece.org/pxweb/?lang=1), and World Bank (http://data.worldbank.org/indicator), from 1990 to 2010^2^ (OECD, 2014; United Nations Economic Commission for Europe, 2014; World Bank, 2014). Across the nine indicators, average ecological correlation with time (Hamamura, 2012) was *r* = 0.94 (*p* < 0.05), suggesting that these variables are changing worldwide with time.

The change stimulated national development in many countries, fulfilling our needs of the modern life style (Inglehart and Baker, 2000). However, during the same period, many developed countries have also been caught up in waves of recession, leading to disparity in the population. During this period, worldwide economic stagnation occurred in East Asian countries in early 1990s, and in European countries during the late 2000s. There were some serious health consequences for marginalized group, such as unemployed, uneducated, or poor individuals (Stuckler et al., 2009) during the time. However, economic turmoil may also affect the health of population at large, via the change in peoples' socio-demographic conditions.

### Psychological consequences of change

Historically, the socio-demographic change toward *Gesellshaft* secured free market and wealth leading to material possession and happiness (Diener, 2000; Inglehart et al., 2008). However, since socio-demographic change, by definition, takes place first in the external environment, it always precedes psychological change (Bardi and Goodwin, 2011). Because human cultural adaptation is built even at the level of automatic behaviors learned in accustomed daily activities (Kitayama and Park, 2010), change in the socio-demographic context would impose an implicit challenge to the efficiency of acquired pattern of behaviors. If acquired behaviors were rendered useless to pursue valued goals in life (Oishi et al., 1999b), our well-being would be compromised. In other words, our health is usually sustained by the culture we live in Diener (2000), Draguns and Tanaka-Matsumi (2003), and Triandis (2007), but for this very reason, socio-demographic change undermining the original can cause mental health problems in the population (Kashima, 2013), by creating unaccustomed mandates (Kitayama et al., 2009).

If the transition had been carried out smoothly, involving well-structured institutions and formal education that supports people to catch up with the new requirements, the change into *Gesellshaft* socio-economic condition may foster independence and also preserve healthy social relations (Kagitcibasi, 2005). However, when the transition is rapid, imminent, allowing no choice but to engage in global competition to survive, general population would fail to adapt. Further, if the original culture was encouraging interdependent ways of life, rapid change into *Gesellshaft* socio-demographic condition might require extra mental effort for the residents (Ogihara and Uchida, 2014).

Possible disadvantages due to such change may range from psychological to social aspects of their health. Specifically, their general health might be compromised because their working conditions become worse in response to economic stagnation. During this period, full-time employees were required to oblige by meeting an intensified workload, and they faced a doubled unemployment rate (Statistics Bureau, 2014). Their life satisfaction might be lowered because subjective well-being can be compromised when valued life domains fail to satisfy individuals due to economic turmoil (Oishi et al., 1999a; Diener, 2000; OECD, 2013). If rapid change requires the members to be alone and exercise new self-ways to be accepted, change might compromise one's self-worth, or the fundamental motivation to belong (Baumeister and Leary, 1995) and one's achievement/acceptance of socially desirable self (Tafarodi and Swann, 1995). Perceived social support might also be compromised as change may increase one's need to relocate and be mobile in the new context (Oishi et al., 2007). Isolated from supportive others, one's perceived social support may decline.

In East Asian countries, the outcome of the change has been observed as a decrease in average psychological health. For example in Japan, people have traditionally regarded interpersonal harmony as their central meaning of happiness (Uchida and Kitayama, 2009). Now they have to leave their close families, elder parent or friends in order to attain new position or decent job, sometimes they are involved in an aggressive competition or candid quarrel against their colleagues in order to compete for status and limited wage. Otherwise, they may give up making new family out of inflated partner choice and economic/business reasons. These events might increase the likelihood of interpersonal concerns or disharmony. Especially for those having interdependent self, the change might undermine their well-being, or severely limit the fulfillment of the motivation to belong.

Given these disadvantages stemming from the glitch at socio-demographic level, some people may survive the influence using specific forms of positive strength. As one of such strengths, we focused on interdependent happiness^3^ (Hitokoto and Uchida, 2014). Interdependent happiness is a collectively shared concept of happiness (Uchida et al., 2014) among the members of interdependent cultures. According to the previous studies (Uchida and Kitayama, 2009; Uchida and Ogihara, 2012), Japanese, compared to European American counterparts, share the meaning of happiness as relational. Whereas this type of happiness is held central to the subjective concept of happiness among certain cultural members, the conception of happiness as relational is considered common across all human being. Because interdependent happiness involves relational harmony, quiescence and ordinariness (Hitokoto and Uchida, 2014), these positive meanings would be more central to residents' well-being in the *Gemeinshaft* societal condition. If the change is an avalanche toward *Gesellshaft* socio-demographic condition, then the traditional interdependent happiness would be a positive buffer against the trend. Therefore, among the traditionally *Gemeinschaft* societal members, the strength of preserving harmony among close others may find the negative influence of change. Particularly, in Japanese case, interdependent happiness will predict health positively while the change will not.

### Aftermath of recession in japan

During the 1990's, Japanese companies faced a collapse of the collectivistic working style, together with the collapse of the real estate *bubble economy*. In the workplace, companies abandoned the lifetime employment and seniority system, in exchange for thrift management^4^. The push for globalizing economy: transparency, international competition, and global communication increased. Even after 2000, the aftermath of this decade and further economic depression delayed Japan's economic recovery. Even more tragic was the fact that, although such change was apparent, formal education continued to encourage *generalists:* all-arounders capable of managing multiple roles in a single company, thus best functional under the long-lasting employment system (Toivonen et al., 2011)^5^, and the major companies preferred naïve freshmen over slightly older professionals as their main labor force.

Coinciding with such tension was an increase in health problems. The decades after 1990s are marked as a time when Japan showed unprecedented deterioration in mental health statistics (Kawakami et al., 2006). Following the *bubble* collapse, over 30,000 workers per year committed suicide. Specifically, the suicide rate of working men in their 30's and 50's increased. The number of outpatients diagnosed with mood disorders doubled between 1990 and 2010. The number of working adults suffering from depression increased, with more people suffering from depression than cancer or diabetes. From the late 1990's to 2010, applications for insurance compensation to workers were dominated by requests from patients with mental disorders (Japanese Association of Schools of Certified Psychiatric Social workers, 2012), and industrial safety and health issues became focused on regulating overwork (Health, labour and welfare statistics associations, 2012). Given this situation, the Ministry of Health, Labor, and Welfare listed mental disorders as one of the big five diseases among Japanese adults in 2011^6^. The decades after 1990 were a time when the lack of fit between traditional interdependence and the requirements of the new liberal, global economic, individualistic environment became apparent, with critical consequences for Japanese health. Kitanaka (2012) vividly portrays these adverse psychological profiles of Japanese workers and a “society in distress” through narrative data.

### Hypothesis

What are the psychological consequences of economic stagnation accompanying demographic changes away from the traditional way of life? In this study, we used a national case approach and focused on Japan as a country where people have traditionally lived in *Gemeinschaft* socio-demographic condition, but now caught up in a rapid change as a consequence of the 1990s (Toivonen et al., 2011).

**Figure 2:**
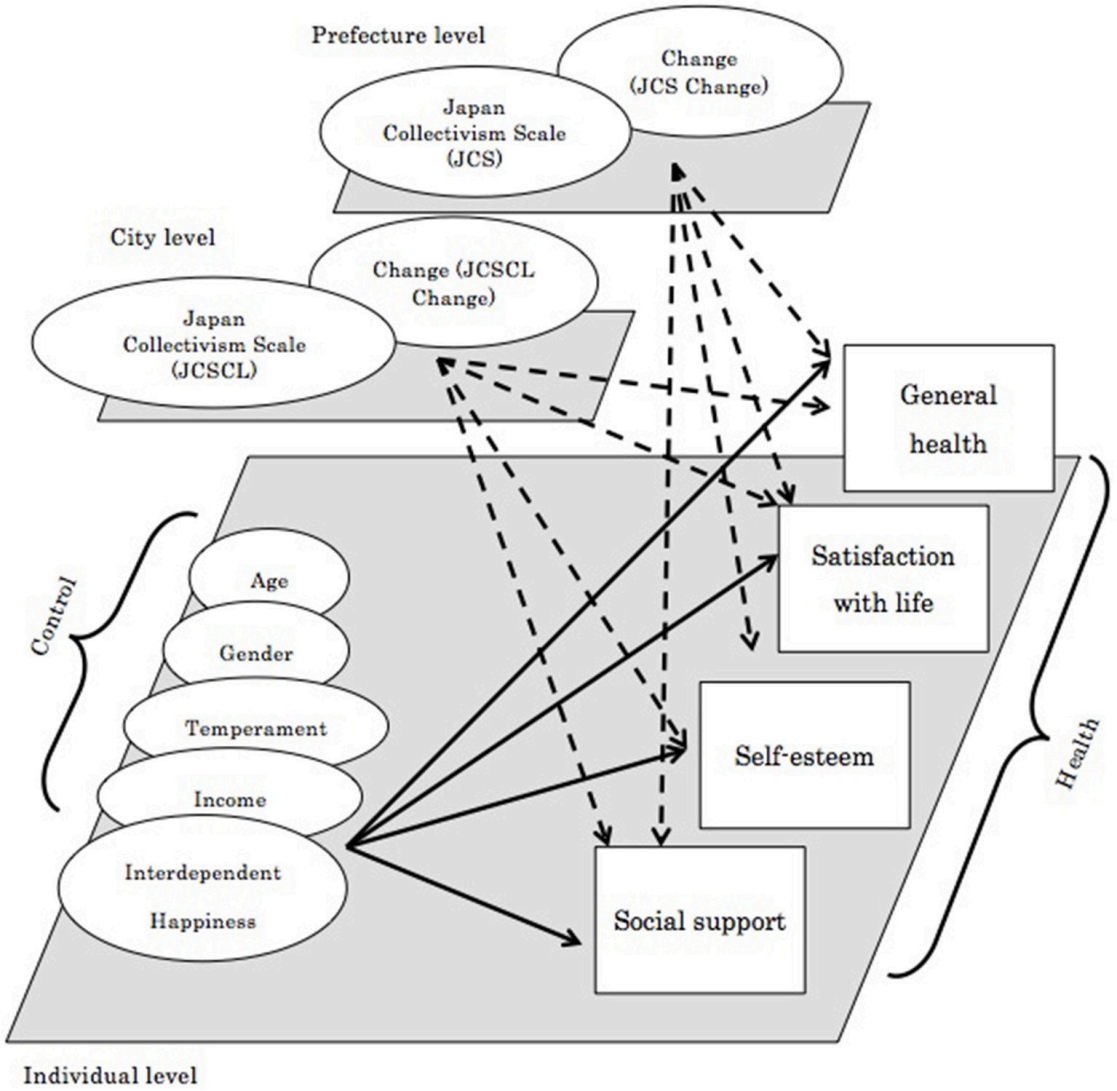
**Hypothesized multi-level model predicting aspects of health from *prefecture*, city, and individual level predictors**. Arrows indicate positive explanatory weights, while broken arrows indicate negative explanatory weights.

Specifically, we hypothesized that among those working adults who underwent large change toward individualistic socio-demographic condition, their psychological health would be more compromised than those who underwent small change (Figure 2). We tested this hypothesis by comparing different regions that systematically differ in the amount of change.

#### Dependent variables

We measured adults' health according to the indicators of (1) general health, (2) life satisfaction, (3) self-esteem, and (4) perceived social support. We hypothesized that the change would negatively predict general health (Hypothesis 1). Also, change will negatively predict life satisfaction (Hypothesis 2), self-esteem (Hypothesis 3), and perceived social support (Hypothesis 4). We divided social support into support provided from one's community and support provided from out of one's community, in order to explore the possible difference between these two. Finally, interdependent happiness would positively predict psychological health (Hypothesis 5).

#### Independent variables

In this study, we use the term “change” to refer to a set of changes in socio-demographic variables related to societal level individualism-collectivism (Vandello and Cohen, 1999), such as family size or divorce. Specifically, the change is captured as (1) a cross-temporal change during 1990–2010, and (2) a change which took place at the level of socio-demographic condition. Therefore, we operationalized change as the difference in scores between two or more fixed points in time (Hofstede, 2001; Zielenziger, 2006; Allen et al., 2007; Inglehart et al., 2008). We examined change using ecological correlations between our indicators discussed below and time (Hamamura, 2012). We measured socio-demographic conditions both at the meso (i.e., prefecture) and micro (i.e., city) levels (Iwata, 2006), and used change at those levels as additional contextual explanatory variable.

In order to compare groups of Japanese adults varying in their degree of change, we used prefecture difference (Plaut et al., 2002; Kitayama et al., 2006). Yamawaki (2012) measured at Japanese prefecture^7^ level: the Japanese Collectivism Scale (JCS). The JCS is a sum of standardized scores of (1) divorce to marriage ratio (reversed), (2) percentage of households with three generations living together, (3) percentage of elderly people (aged 65 + years) living alone (reversed), (4) percentage of nuclear family households (reversed), and (5) percentage of people living alone (reversed) in 2006.

Communities also vary by how people make living within their ecologies (Uskul et al., 2008) and they show sizable group differences (Gordon, 2003; Uskul et al., 2008; Fukushima et al., 2011). We considered worthwhile to examine the impact of change at smaller levels than prefectures from the viewpoint of community-based policy making (Raudenbush, 2003; Uchida and Takemura, 2012). We created a JCS city level variable (JCSCL)^8^ by aggregating the JCS indicators at the city level^9^.

We evaluated societal level explanatory effects after controlling for basic individual difference variables. For those, we controlled for personality traits (Cloninger, 1987), gender (Kashima et al., 1995), age (Matsumoto et al., 1996) and income (Snibbe and Markus, 2005) in the analysis. As for income, considering the gender distribution of labor altering the income opportunity of adults in Japan, we measured own income independent of partner's income, and controlled these two in the analysis.

In order to test our model, we first calculated the extent to which each prefecture and each city have changed using archival data (Study 1). We then used those change scores to explain the health of residents living in diverse areas in Japan using social survey (Study 2).

## Study 1: measurement of prefecture and city level change

First, we examined how JCS (Yamawaki, 2012) changed between 1990 and 2010. Second, JCSCL and their changes were measured. We tested reliability and validity using criteria that are both available at the city level. Because the change after 1990s should have affected both urban and rural regions during this special period of economic disaster and globalization (Norasakkunkit et al., 2012), we expected change in both urban and rural prefectures.

### Method

#### Prefecture change

We collected archival data on five indicators of the JCS (Yamawaki, 2012) for 47 prefectures in Japan from Japan's official census statistics site (http://www.e-stat.go.jp/SG1/estat/eStatTopPortalE.do) at 1990, 1995, 2000, 2005, and 2010. We used JCS at 2010 as a prefecture level Individualistic-Collectivistic socio-demographic condition score in this study^10^. The five indicators included divorce to marriage ratio, percentage of households with three generations living together, percentage of elderly people (aged 65 + years) living alone, percentage of nuclear family households, and percentage of people living alone (reversed). A total of 1,175 (5 indicators × 47 prefectures × 5 time points) data points were collected. We calculated internal consistencies for every sampled year. We aggregated the standardized subtraction scores of each indicator between 2010 and 1990 as an indicator of change.

#### City change

Five indicators of JCS were collected at the city level (*n* = 1750) from the same archive^11^. We tested reliability of the JCSCL by calculating internal consistency for every sampled year. We evaluated validity of the JCSCL using correlations with the city level variables of (1) taxable income, (2) percentage of adults working in primary industry, (3) percentage of adults working in tertiary industry, (4) move-in rate, and (5) move-out rate, following past research (Hofstede, 2001; Yamawaki, 2012). Then, we calculated cross-temporal change of JCSCL.

### Results and discussion

#### Reliability of the JCS (prefecture level)

The reliability (internal consistency) of the JCS indicators in each sampled year was marginal to acceptable (0.68 < α < 0.84) as in the original study (Yamawaki, 2012)^12^.

#### Changes of prefecture socio-demographic condition

**Figure 3:**
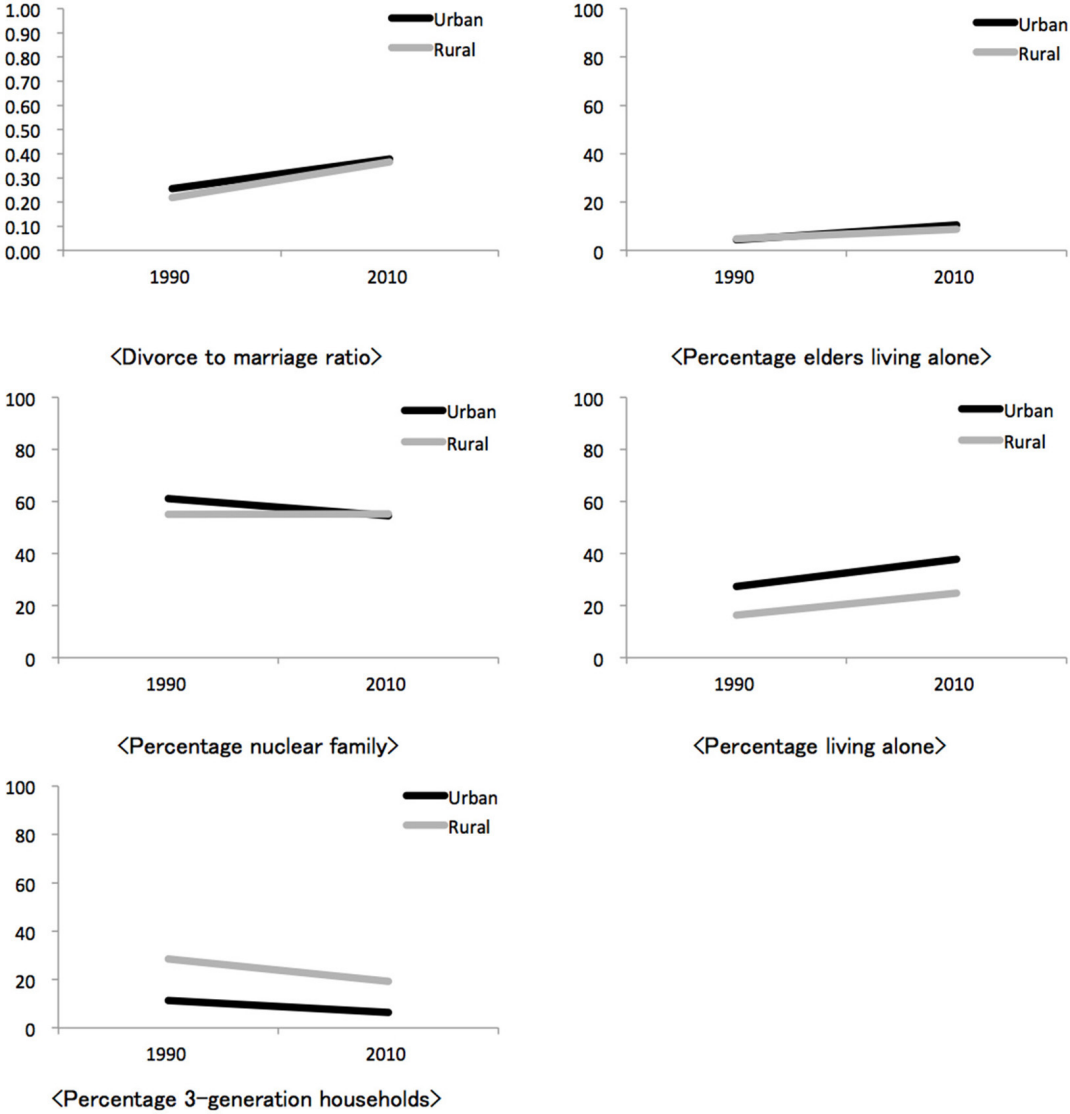
***Prefecture* change in the indicators of the JCS during 1990–2010**. Lines represent averages of either most urban (*Tokyo, Osaka, Hokkaido, Fukuoka*) or rural (*Yamanashi, Wakayama, Yamagata, Saga*) prefectures that are geographically apart from each other.

The first column of Table 1 shows the correlation between socio-demographic indicators and time at *prefecture* level. In four out of five indicators, their changes were in the direction of increased individualistic socio-demographic condition. However, for percentage of nuclear family, the trend showed reversed tendency, indicating marginal change toward collectivistic socio-demographic condition. Closer examination revealed this reversed correlation to be limited to urban prefectures (Figure 3). This indicator change may be related to the population increase in urban areas, however, in this study we followed Yamawaki (2012) to use this as an indicator.

**Table 1:** **Correlation coefficients between socio-demographic indicators and time**.

	**Correlations**			
	**Between prefecture average and time**	***p***	**Between city average and time**	***p***
Divorce to marriage ratio	0.952	*	0.961	**
Percentage elder living alone	0.999	***	0.999	***
Percentage nuclear family	−0.868	†	0.603	
Percentage living alone	0.997	***	0.999	***
Percentage three-generation households	−0.999	***	−0.999	***

*** *p < 0.001*,

** *p < 0.01*,

* *p < 0.05*,

† *p < 0.1*.

Figure 3 shows representative change for each indicator. The urban and rural groupings were made based on the number of households and gross domestic prefecture income. Within that variation, the top four urban and rural prefectures (urban: *Tokyo, Osaka, Hokkaido, Fukuoka*, and rural: *Yamanashi, Wakayama, Yamagata, Saga*) that (1) are located apart within each group and (2) have closely located pairs across groups were selected, and their JCS scores were averaged in 1990 and 2010, respectively. Both urban and rural prefectures showed trends toward individualistic socio-demographic condition, except for percentage of nuclear families in urban areas.

The JCS and its change score correlated positively (*r* = 0.34, *p* < 0.05), indicating prefectures with collectivistic socio-demographic condition showed more change during this period.

**Figure 4:**
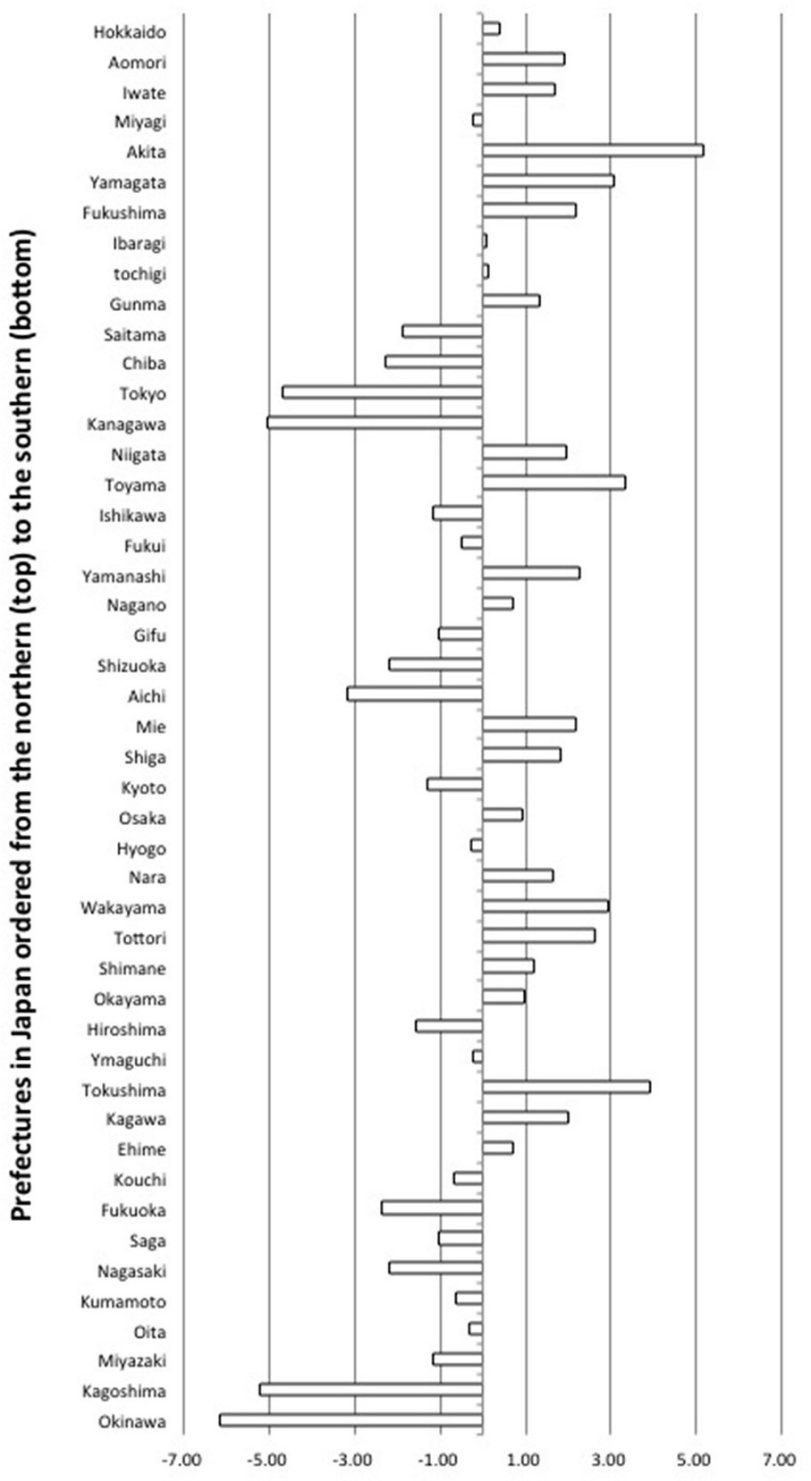
**Aggregated standardized change for each prefecture (*N* = 47)**. Zero indicates average amount of relative change during 1990–2010 across 5 indicators of individualistic socio-demographic condition. Minus and plus figures indicate below and above average change, respectively, during this period.

In Figure 4, a bar describes the sum of standardized change across indicators for one prefecture, or the degree to which a prefecture changed into individualistic socio-demographic condition in comparison to others. The rate of change differed across prefectures^13^.

#### Reliability and validity of the JCSCL (city level)

**Figure 5:**
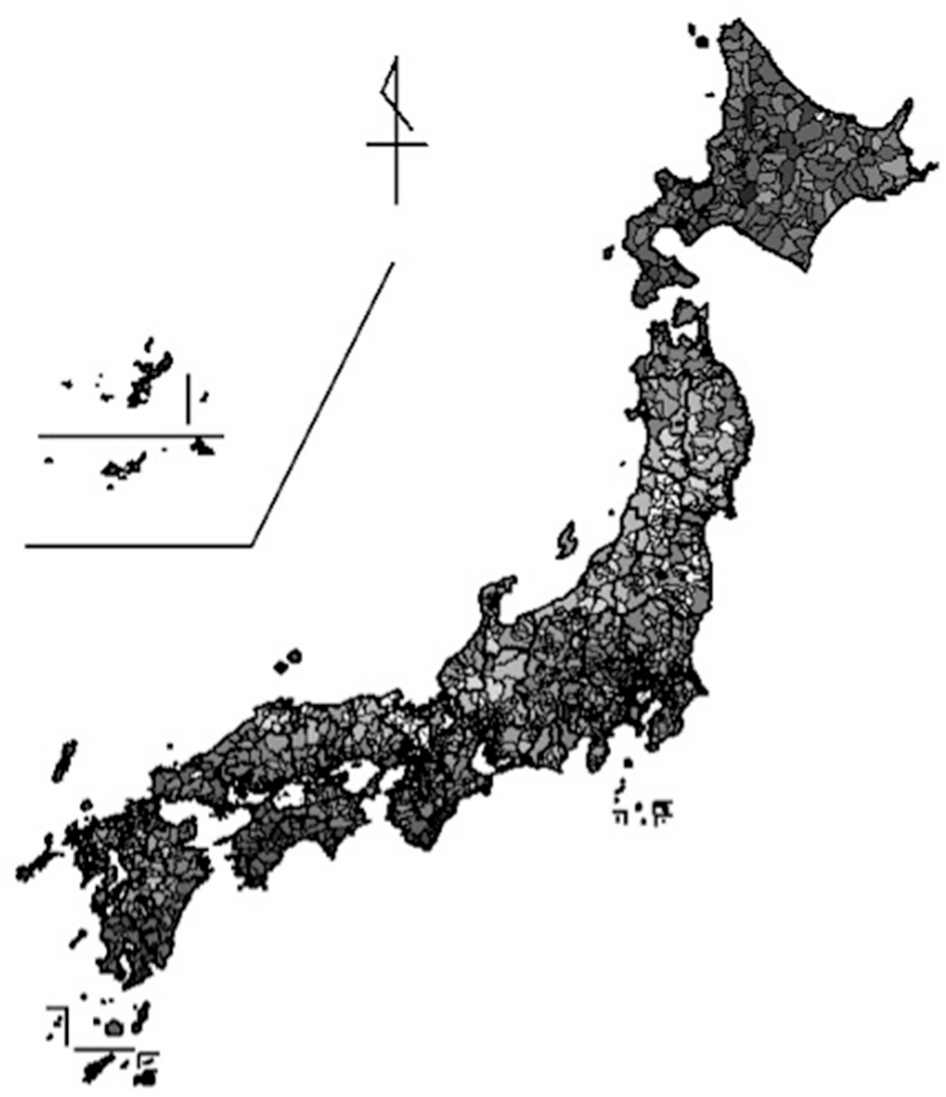
**City level distribution of Japanese Collectivism Scale calculated at city level**. The lighter color indicates higher collectivistic socio-demographic condition of the city in Japan.

As in Yamawaki (2012), the JCSCL also showed geographic similarity between the neighbors; for example, those cities located around the *Sea of Japan* coastal area are generally collectivistic, pointing to the possibility that city level socio-demographic condition is clustered geographically (Figure 5). The reliability of the JCSCL was lower (0.39 < α < 0.62) than that of the JCS in every sampled year. Similar to the JCS, there was also a declining trend of the reliability over time. The validity coefficients were *r* = −0.11 (*p* < 0.001) with income, *r* = 0.11 (*p* < 0.001) with workers in primary industry, *r* = −0.44 (*p* < 0.001) with workers in tertiary industry, *r* = −0.36 (*p* < 0.001) with move-in rate, and *r* = −0.40 (*p* < 0.001) with move-out rate, all in expected directions. These indicate reasonable relationships between criteria variables and the JCSCL; yet, they show lower validity coefficients than those of the JCS (Yamawaki, 2012)^14^.

#### Changes of city socio-demographic condition

The second column of Table 1 shows the correlation between socio-demographic indicators and time at city level. All indicators changed in the direction of increased individualistic socio-demographic condition, with percentage of nuclear family showing weaker correlation than other indicators.

The changes in the JCSCL scores also varied between communities^15^. The JCSCL and its change score were very weakly correlated (*r* = 0.16, *p* < 0.001), or in other words, city level socio-demographic condition was weakly related to its change during this period.

In Study 1, we measured socio-demographic indicators by prefecture and city, and have observed variation in change. Utilizing this variation as a comparison ground, we conducted Study 2 to examine if Japanese subjective health would differ significantly depending on the degree of change.

## Study 2: explaining adult health from change, prefecture/city socio-demographic conditions, and individual differences

We surveyed Japanese adults living in various prefectures in Japan, and tested the hypothesis model (Figure 2).

### Method

#### Participants

The survey was conducted as a part of a faculty development program to randomly sample graduates of a private university in *Hyogo* prefecture, one of the prefectures with low score of JCS (Yamawaki, 2012), with students from relatively high familial socio-economic status and high school educational attainment. From the 29,507 graduates who listed their names on the alumni list, 7,628 currently living in Japan were randomly selected as the survey target. They were stratified so as to include every major in the university, and included graduates from every year between 1938 to 2008. Response rate of the survey was 47.2%.

Within the 3,248 collected, we discarded those participants who (1) belonged to a prefecture with less than 10 participants in each prefecture or city, and (2) those who belonged to an outlier city in terms of the distribution of JCSCL, by 2 standard deviations distance from the mean. The selection resulted in 2,343 participants (1617 males, 709 females, 17 unknown, mean age 51.49, *SD* = 18.21) who were living in 53 communities across 17 prefectures (sample distribution is shown in the Supplementary Material, Appendix 1 and 2)^16^.

#### Measures

For the explanatory variables, we used the Interdependent Happiness Scale (IHS; Hitokoto and Uchida, 2014), which consists of 9 items asking whether participants experience happiness in their interpersonal relationships. Items included statements such as, “I believe that I and those around me are happy,” or “I feel that I am positively evaluated by others around me.” Participants responded using a 5-point (1: Completely agree—5: Completely disagree), Likert-type scale. The regression weight for this scale score on health measures would respond to our hypothesis 5.

We used the Temperament and Character Inventory (TCI: Cloninger, 1987; Kijima et al., 2000) to measure the personality difference. Because of limited space for questions in the joint survey, we used 2 or 3 representative items that have shown high loadings in the past Japanese study, to tap each of the 4 dimensions of temperament. Examples of TCI items were, “I like to use money rather than save it (novelty seeking),” or “I often get nervous or worried when doing new things I am not familiar with (harm avoidance).” Participants responded using a 4-point (1: Completely agree—4: Completely disagree), Likert-type scale.

We also asked about participants' annual income, including the annuity, from both the self and the partner as a measure of economic condition. Because Japanese adults have large gender differences in working roles, we asked these two separate questions and analyzed by controlling for gender. We asked participants to choose one option from 0: no income, 1:–3.99 million Yen (i.e., approximately less than $40,000), 2: 4–5.99 million Yen ($40,000–60,000), 3: 6–7.99 million Yen ($60,000–80,000), 4: 8–9.99 million Yen ($80,000–100,000), 5: more than 10 million Yen (more than $100,000), separately for themselves and their partner if they were married.

To test our hypothesis 1, we used the General Health Questionnaire (GHQ; Goldberg and Hillier, 1979; Nakagawa and Daibo, 1985). Examples of items from this questionnaire are, “I couldn't sleep well, because I had worries,” or “I was able to have more fun in my daily life than usual.” Participants rated how they felt/experienced each day using a 4-point (1: I did/didn't—4: I didn't/did, depending on the item) Likert-type scale.

To test hypothesis 2, we used the 5-item Satisfaction with Life Scale (SWLS; Diener et al., 1985) as a measure of subjective well-being. The SWLS consisted of items such as, “I am satisfied with my life as a whole,” and participants responded as to whether they would agree to each statement using a 7-point (1: Strongly disagree—7: Strongly disagree) Likert-type scale.

To test hypothesis 3, we used 2 indicator items tapping self-liking (“I like myself”) and self-competence (“I am talented”) aspects of self-esteem (Tafarodi and Swann, 1995; Sakurai, 2000) as a measure of global self-evaluation (Pallant and Lae, 2002; Johnson, 2004). Participants rated how the items applied to themselves using a 5-point (1: Completely agree—4: Completely disagree), Likert-type scale.

To test hypothesis 4, we asked about one's perceived social support, both in and outside of one's community, since working and retired adults might have significant others in or outside of the area in which they lived. The question was, “To what extent do you have friends/acquaintances whom you can help or talk to each other?” We asked them to rate separately, “friends/acquaintances in the community (*Chi-iki* in Japanese)” and “friends/acquaintances out of the community,” on a 3-point scale (1: I have many, 2: I have a few, 3: I have none).

Before analysis, scores of GHQ and social support were reversed so that a higher score means better health. For the rest of the scales, higher score indicates better health. Descriptive statistics and reliabilities of the scales are listed in Table 2. Except the four TCI subscales, which showed marginal reliabilities (0.52 < α < 0.64), other measures showed acceptable levels of reliability. Explanatory variables were weakly correlated with each other (Table 3).

**Table 2:** **Descriptive statistics and reliabilities of the measures/items**.

**Measures**	***Min***	***Max***	***M***	***SD***	***n* of items**	**α**
**EXPLANATORY VARIABLES**						
Novelty seeking (TCI^a^)	3	12	6.80	1.56	3	0.52
Harm avoidance (TCI)	3	12	8.18	1.49	3	0.58
Reward dependence (TCI)	3	12	8.91	1.50	3	0.64
Persistence (TCI)	2	8	5.57	1.15	2	0.64
Interdependent Happiness (IHS)	9	45	33.33	5.66	9	0.90
**EXPLAINED VARIABLES**						
General health (GHQ)	0	12	9.56	2.84	12	0.85
Satisfaction with life (SWLS)	5	35	22.37	5.55	5	0.90
Self-esteem	2	10	6.96	1.37	2	0.75
Perceived social support in community	1	3	0.92	0.64	1	–
Perceived social support out of community	1	3	1.21	0.57	1	–

a *TCI, Temperament and Character Inventory*.

**Table 3:** **Correlations among individual level explanatory variables**.

	**Age**	**Gender**	**TCI novelty seeking**	**TCI harm avoidance**	**TCI reward dependence**	**TCI persistence**	**Own income**	**Partner's income**	**Inter-dependent happiness**
Age									
Gender^a^	−0.36^**^								
TCI^b^ Novelty Seeking	0.03	−0.01							
TCI Harm Avoidance	−0.12^***^	0.16^***^	−0.09^***^						
TCI Reward Dependence	−0.10^***^	0.08^**^	−0.02	0.02					
TCI Persistence	−0.04^*^	0.04^†^	−0.25^***^	−0.14^***^	0.19^***^				
Own income	0.14^***^	−0.44^***^	0.00	−0.17^***^	−0.02	0.02			
Partner's income	0.00	0.41^***^	−0.03	0.04^†^	−0.01	−0.01	−0.16^***^		
Interdependent Happiness	0.12^***^	0.08^***^	−0.06^**^	−0.28^***^	0.10^***^	0.17^***^	0.09^***^	0.18^***^	

a *Gender was coded as 1 = male, 2 = female*.

b *TCI, Temperament and Character Inventory*.

*** *p < 0.001*,

** *p < 0.01*,

* *p < 0.05*,

† *p < 0.1*.

#### Analysis

For each of the dependent measures, we used the same set of explanatory variables (Figure 2). In the model, the first level explanatory variables were, age, gender, four temperaments (i.e., novelty seeking, harm avoidance, reward dependence, and persistence), and interdependent happiness. Second level explanatory variables were the 53 JCSCL scores assigned for available communities/cities and the degree of their change from 1990 to 2010. Third level explanatory variables were the 17 JCS scores assigned for available prefectures and the degree of their change from 1990 to 2010. First and second level explanatory variables were group mean centered. The higher level explanatory variables were modeled to explain the intercept of their lower level. Therefore, the model tested would be described as follows.

$$\begin{array}{l} \text{Level-1 Model}:\\ \text{ Health measure }(\text{i}.\text{e}.,\text{GHQ})=P0\\ \;+\;P1\times (\text{Age})+P2\times (\text{Gender})\\ \;+\;P3\times (\text{TCI Novelty seeking})\\ \;+\;P4\times (\text{TCI Harm avoidance})\\ \;+\;P5\times (\text{TCI Rewarddependence})\\ \;+\;P6\times (\text{TCI Persistence})\\ \;+\;P7\times (\text{Own income})\\ \;+\;P8\times (\text{Partner}\prime \text{s income})\\ \;+\;P9\times (\text{Interdependent happiness})+e\\ \text{Level-2 Model}:\\ \;P0=B00+B01\times (\text{JCSCL})+B02\times (\text{JCSCLChange})+r\\ \text{Level-3 Model}:\\ \;B00=G000+G001\times (\text{JCS})+G002\times (\text{JCSChange})+u \end{array}$$

Because each participant was nested within prefectures and cities, we first examined how much variation in our dependent variables would be accounted for by group differences *per se* using intra-class correlation (*ICC*). We used the HAD program (Shimizu et al., 2006) to calculate the *ICC* and conventional *F* ratio comparing the variance within and between the prefectures for each of the dependent variables, separately for prefecture and city (Supplementary Material, Appendix 3). Given the fact that the typical *ICC* in applied research is around 0.02–0.22 (Cheung and Au, 2005), there was very little group difference between prefectures or cities. This would indicate that when using self-reported health data among Japanese adults, there are almost negligible differences between prefectures and cities compared to the individual variation within them. With these small effect sizes in mind, we conducted Hierarchical Linear Modeling using HLM7 (Raudenbush and Bryk, 2002).

### Results and discussion

The results show strong and stable relationships between individual difference variables and health, and small but significant effects of change (Table 4). This indicates that Japanese health is explained largely by individual level factors, and to a lesser extent, by the temporal change of socio-demographic conditions at both *prefecture* and city levels.

**Table 4:** **Effects of explanatory variables on mental health measures**.

**Explanatory variables**	**Parameter**	**Explained Variables**														
		**General health**			**Satisfaction with life**			**Self-esteem**			**Social support in community**			**Social support out of community**		
		***B***	***SE***	***p***	***B***	***SE***	***P***	***B***	***SE***	***p***	***B***	***SE***	***p***	***B***	***SE***	***p***
**PREFECTURE LEVEL EFFECTS**																
JCS^a^	G001	−0.03	0.04		−0.04	0.07		0.00	0.02		0.02	0.01	†	−0.02	0.01	*
JCS Change^b^	G002	0.01	0.04		−0.21	0.07	*	−0.03	0.02	†	0.00	0.01		−0.02	0.01	**
City level effects																
JCSCL^c^	B01	0.08	0.13		0.03	0.27		−0.17	0.06	**	0.06	0.03	*	−0.01	0.02	
JCSCL Change^d^	B02	−0.05	0.08		−0.02	0.16		−0.04	0.03		−0.02	0.02		0.00	0.02	
**INDIVIDUAL LEVEL EFFECTS**																
Age	P1	0.02	0.00	***	−0.02	0.01	**	0.00	0.00	**	0.00	0.00		0.00	0.00	***
Gender^e^	P2	−0.35	0.14	*	−0.42	0.26		0.03	0.07		−0.01	0.04		0.02	0.03	
TCI^f^ Novelty seeking	P3	−0.02	0.03		0.07	0.06		0.00	0.02		0.02	0.01	*	0.02	0.01	*
TCI Harm avoidance	P4	−0.29	0.04	***	−0.36	0.06	***	−0.15	0.02	***	−0.05	0.01	***	−0.03	0.01	***
TCI Reward dependence	P5	−0.13	0.03	***	0.03	0.06		0.05	0.02	**	0.04	0.01	***	0.04	0.01	***
TCI Persistence	P6	−0.01	0.05		0.36	0.08	***	0.18	0.02	***	0.01	0.01		0.04	0.01	***
Own income	P7	−0.04	0.04		0.12	0.06	†	0.01	0.02		−0.05	0.01	***	0.00	0.01	
Partner's income	P8	−0.04	0.04		0.16	0.07	*	−0.01	0.02		0.02	0.01	*	−0.01	0.01	
Interdependent Happiness	P9	0.23	0.01	***	0.59	0.02	***	0.10	0.00	***	0.02	0.00	***	0.01	0.00	***

a *Japanese Collectivism Score. Higher the score, the more prefecture level collectivistic socio-demographic condition*.

b *Change of Japanese Collectivism Score. Higher the score, the more prefecture change toward individualistic socio-demographic condition*.

c *Japanese Collectivism Score at city level. Higher the score, the more city level collectivistic socio-demographic condition*.

d *Change of Japanese Collectivism Score at city level. Higher the score, the more city change toward individualistic socio-demographic condition*.

e *Gender was coded as 1 = male, 2 = female*.

f TCI, Temperament and Character Inventory

*** *p < 0.001*,

** *p < 0.01*,

* *p < 0.05*,

† *p < 0.1*.

In addition to such a general association, there were differences in the combinations of these societal variables affecting specific health-related variables (Table 4). Contrary to our hypothesis 1, general health was not explained by change, but was explained more by individual age, gender, harm avoidance and reward dependence, and interdependent happiness.

Supporting our hypothesis 2, satisfaction with life was low among those who live in changed prefectures (i.e., negative weight of JCS change on satisfaction with life in Table 4), but city level change did not show the same weight.

Supporting our hypothesis 3 weakly, self-esteem also tended to be low in changed prefectures (i.e., marginally negative weight of JCS change on self-esteem). Negative weight of JCSCL on self-esteem might reflect a relationship between collectivism and low self-esteem (Heine et al., 2001). However, these predicted effects were smaller than the individual differences in harm avoidance, persistence and interdependent happiness, suggesting that subjective well-being and positive self-regard are largely predicted by individual emotional predispositions and interdependent happiness, if within-country difference is concerned.

As for hypothesis 4, social support in one's community was not explained by change, but tended to be higher among collectivistic socio-demographic prefectures, and high among such communities (i.e., marginally and significantly positive weight of JCS and JCSCL on social support in community). Social support out of one's community was negatively explained by *prefecture* change (i.e., negative weight of JCS change on social support out of community), suggesting deterioration of distal support during this period. Collectivistic socio-demographic prefectures, but not cities, showed lower support from outside of one's community (i.e., negative weight of JCS on social support out of community). Again, individual level factors predicted social support significantly, and harm avoidance and reward dependence, as well as interdependent happiness, were the significant predictors of social support.

Supporting our hypothesis 5, in all health measures examined, interdependent happiness showed positive explanatory weight. This is an indication that interdependent happiness—relational harmony, quiescence, and ordinariness among Japanese adults—is an integral part of their health, independent of change during this period.

### General discussion

In this study, we examined the relationship between socio-demographic change and health, using Japanese adults living in various prefectures and cities. We tested whether the change pertaining to the increase of individualistic socio-demographic condition is related to health during 1990–2010, when the Japanese economy and culture was undergoing massive economic turmoil (Norasakkunkit et al., 2012). As a result, most of our hypotheses were supported and some were not. That is, hypotheses 2 regarding the negative prediction of life satisfaction was supported and hypothesis 3 on self-esteem was weakly supported. Also, hypothesis 5 which stated the positive prediction of interdependent happiness onto health measures was supported. Regarding hypothesis 4 which stated the negative impact of change onto perceived social support, showed mixed results. That is, change at the prefecture level did relate to less perceived social support out of one's community, but did not relate to that of inside of one's community. Finally, hypothesis 1 with regard to the negative prediction of change onto general health was not supported. Considering the overall pattern of results, the consequences of economic turmoil might be related to self-evaluative and the social domain of health.

#### Pan-cultural implications

The present findings support the argument that rapid socio-demographic change can be related to psychological difficulties (Norasakkunkit et al., 2012). We may be able to extend such argument by focusing on the socio-demographic change and consider adult health. Also, the result suggests the importance of cultural well-being (Uchida and Ogihara, 2012) as a psychological strength, and basic individual difference in sustaining the health, since the best predictors of health were interdependent happiness and personality^17^. If individual differences are in turn sustained by the larger national culture (Kitayama et al., 2007; Hitokoto and Uchida, 2014), then both personal and social explanations may need to be used together (Iwata, 2006) to explain adult health. Future studies will need to clarify more concrete processes of this contextual aspect of our health, such as how, and what events surrounding the individual would mediate this cross-level impact on health.

#### Implications for japanese culture

Current results showed that the socio-demographic change which took place during this period was negatively related to the Japanese well-being. Specifically, in more changed prefectures, such as *Akita*, *Tokushima* or *Toyama* prefectures, adults are more unsatisfied with their life, tend to have lower self-esteem, and less social support from out of one's community compared to adults in other prefectures that underwent smaller amounts of change. Additionally, *prefecture* and city level collectivistic socio-demographic condition was positively related to social support in one's community. Our study also resonates with the findings on community strength in Japan (Fukushima et al., 2011), in that both types of research focus on societal level resources above and beyond the individual, that in turn sustain residents' health. Future studies need to disentangle the correlates between the socio-demographic condition we used and other societal resources in Japan.

#### Limitations of the study

In order to disentangle the relationship between change and health one step further, more effective research design would include collecting cross-sectional data measuring a set of health and personality variables from the same, as well as age-diverse participants over time. Because our individual data came from a single shot survey, the results may differ when within-subject data are used to control for participants' past health, as well as past individual level values or personality. By using a large data set involving cohort design (Ryff, 2008) we would be able to evaluate the effect of change on the deterioration of health in individuals.

In our analytical model we assumed forcefully that individuals had not lived in different prefectures or cities, which is probably not the case. In the joint survey we used in Study 2, we additionally measured individuals' original prefecture defined as the prefecture participants had lived longest before reaching 15 years old. The results we found for the living prefecture did not emerge when participants are grouped into their original prefecture. Even though Japanese do not relocate as often as Americans on average (Kitayama et al., 2006), residential moves strongly affect how individuals construe the self and positive affective experience (Oishi et al., 2007), regardless of country (Oishi et al., 2009). It is vital for future research to disentangle the factor of residential moves from what we found regarding *prefecture* and city socio-demographic conditions, and test if our findings are robust after controlling for individual moves. In doing so, it might also be wise to take into account age at the time of the move (Cheung et al., 2010) as well as the specific combinations of the locations of moves.

Further, considering the time span of change in the socio-demographic condition in Japan (Hamamura, 2012), one can also argue that Japanese health problems might have had roots long before the 1990. Locating the temporal cause may be extremely difficult without the guide of any theory, but finding an auto correlation between variables (Varnum and Grossman, 2013), or comparing the same set of variables across different time periods (Oishi et al., 2011) might be an interesting investigation.

It must be noted that our analysis contained *prefecture* scores that showed negligible *ICC*. Especially, our city level socio-demographic condition showed low reliability, and this may question the reliability of our final outcomes. We note that removing city level factors from the HLM model did not alter our overall findings for other levels. If we were able to find different dimensions that successfully describe cross-regional differences in health, it might override the effect of individual differences we observed in this study. It could also be the case that simply applying national level cultural dimensions to lower level socio-demographic conditions may not be useful to describe health. National level explanations and their lower counterparts may better be kept separate, so that we can theorize and search for more reliable *prefecture*, or city, or even lower dimensions of sub-cultures that provide successful contextual explanations that are open to, therefore more suitable to, describing practical problems and considering remedies.

Finally, we did not model interactions between different levels in our analysis. That is, there still remains a possibility that the explanatory effects of individual level variables on health are systematically different across regional contexts. Such interactions can be hypothesized when we are able to deduce intricate hypotheses regarding correlational differences across regional as well as temporal contexts.

## Summary

In this study, we started from the research question: what are the psychological consequences of economic stagnation accompanying demographic changes away from the traditional way of life? Based on this, we prepared five hypotheses testing negative relationships between *prefecture* and city level change toward individualistic socio-demographic condition from 1990 to 2010, with five variables of individual health. The results overall suggest marginal to significant weights of change on life satisfaction, self-esteem, and social support out of one's community. We also found positive effects of *prefecture* or city level JCS (Yamawaki, 2012) on perceived social support in one's community, as well as large weights of interdependent happiness (Hitokoto and Uchida, 2014) and temperament at the individual level. Future studies should discern further which time period of change or level of socio-demographic condition would truly affect our important life outcomes, using cross-sectional and more representative samples and taking into account the mobile nature of recent Japanese life.

### Conflict of interest statement

The authors declare that the research was conducted in the absence of any commercial or financial relationships that could be construed as a potential conflict of interest.

## Footnotes

^1^A surge in the market caused by speculation regarding a commodity which results in an explosion of activity in that market causing vastly over inflated prices. The prices are not sustainable, and a *bubble* is usually followed by a crash in the affected sector. In the Japanese case, land prices first inflated and then crashed.

^2^We selected nine variables based on Greenfield (2013) and Hofstede (2001), as indicators of *Gesellshaft* or socio-demographic conditions (Greenfield, 2013) related to individualism (Vandello and Cohen, 1999; Yamawaki, 2012) of the country.

^3^Hitokoto and Uchida (2014) developed the interdependent happiness scale (IHS), which consists of items pertaining to relational harmony, quiescence, and ordinariness, distinguishable from other types of happiness.

^4^In households, single living and divorce increased, and the average number of family members decreased rapidly during this period (Statistics Bureau, 2010).

^5^Such a mysterious mixture of change and tradition can be perceived at a glance in a single workplace where elders endorse a traditional value orientation in contrast to the young (Kawakami and Glazer, 2010), or in the fact that elders are more collectivistic (Matsumoto et al., 1996).

^6^In addition to these mental health issues, other societal problems also increased, such as social withdrawal, lowering of the birth rate, substance addiction, pathological gambling, school bullying, and teachers' mental issues, juvenile suicide, unemployment, economic disparity and the “lonely death (*Ko-doku-shi*)” of elders (Japanese Association of Schools of Certified Psychiatric Social workers, 2012).

^7^Prefectures in Japan are the officially defined local governments that support citizens' efficient political administration and development of an area. They originate from the boundaries between local warlords during the Edo era that lasted for 300 years, until late 19th century. They are treated as inclusive geographic regions that have distinctive natural and unique historical aspects such as resources, main industries, or dialects.

^8^Based on the argument by Na et al. (2010), and Hofstede et al. (2010), we expected systematic differences in the reliabilities of JCS and JCSCL. Using various behavioral measures that replicated Japanese and European American differences, Na et al. (2010) found these measures to be uncorrelated with each other within nation, but correlated at the national level, demonstrating a cross-level difference in the reliability. Hofstede et al. (2010) also used his value survey module in a survey of Brazilian states, and poorly replicated his cross-national level factor structure at the cross-state level. If these studies are showing that national level indicators do not always hold at lower levels of analysis, collective behaviors such as percentage of people living with parents or divorce rate may be strongly correlated with each other, thus reliable, at most at the national level (i.e., individualism score and living with parents *r* = −0.67; and divorce rate *r* = 0.76, Hofstede, 2001). But, once the same societal behaviors are collected at the lower *prefecture* or city level, the same indicators may lose their reliability accordingly because of the loss in “appropriate” level of analysis. Therefore, if we calculate JCSCL at the city level, we should expect even lower reliability and validity than Yamawaki (2012)'s JCS. Based on this reasoning, we tested the reliability and validity of JCS and JCSCL, hypothesizing that the former would be larger than the latter.

^9^Cities in Japan are officially the most basic individual unit of local government, and their range is defined by population size and concentration of residences. Normally a city is led by a mayor, and each unit has its own board of education to administer formal schooling. While prefecture is rooted in a historical and geographical divide, the city is one of the closest and most rooted living units for residents in Japan. Traditionally, neighbors sharing the same city had specific as well as unwritten norms to oblige and help each other on ceremonial occasions such as marriages and funerals, in exchange for a daily commitment to district matters through assigned roles for each house. Even today, households often have some assigned roles in city affairs such as gatherings or athletic meetings, and people interact face-to-face with their neighbors and most children are formally educated at local schools located within each city.

^10^JCS calculated at 2010 strongly correlated with JCS at 1990 (*r* = 0.96, *p* < 0.001), as well as JCS at 1980 (*r* = 0.98, *p* < 0.001). The results would indicate, that even though absolute value has changed, the relative position of *prefecture*s on JCS was similar way before the target time period.

^11^Because “percentage of three generation households” was not available at this level from the same archive, we excluded it from 1990 and 1995, allowing 4 items instead of 5, in constructing JCSCL in the 1990's. This item reduction resulted in systematic lowering of the reliability of the JCSCL during the 1990's.

^12^The reliability tended to decrease along with time, suggesting more unreliable measurement of JCS in recent years.

^13^*Akita*, *Toyama*, or *Tokushima*, prefectures that had large population and had neighboring prefectures that were more urban, seem to have changed greatly during the period. In turn, *Okinawa and Kagoshima*, the most southern prefectures in Japan, showed smallest change. In addition, *Tokyo* and *Kanagawa*, two of the most modern prefectures, showed the least change during the period.

^14^Validity coefficients of the national cultural indicators might greatly attenuate at lower levels such as cities. A similar case was found when national level indictors were collected to examine city level difference in U.K. (0.05 < α < 0.47; Gordon, 2003). However, such low level of reliabilities throughout different years would indicate that either (1) the concept of Individualism-Collectivism (I-C) is not best applicable to describe city differences, or (2) measurement error is unavoidable when locating city level I-C using this specific set of indicators and we need to come up with different indicators at lower levels (i.e., cities).

^15^For example, the largest change toward individualistic socio-demographic condition took place in *Ri-shiri* town at the northern island of *Hokkaido* prefecture, where single living increased from 10% to 40% within two decades. In turn, the largest collectivistic change was observed in *A-guni* village in *Okinawa* prefecture, where the percentage of elders living alone decreased from 27.23% to 16.67% within the period.

^16^Participants represented the student distribution of the majors in this university (school of theology: 0.3%; literature: 20.0%; sociology: 14.1%; law: 18.4%; economics: 19.6%; commercial science: 19.1%; engineering: 3.1%; policy: 1.8%). The occupation they held the longest during their lifetime varied (labor job: 0.3%; skilled worker: 0.5%; sales person: 16.2%; security staff: 0.3%; office worker: 19.3%; engineer: 3.2%; specialized skill worker: 6.1%; middle management; 21.2%; upper management: 4.9%; self-employed worker: 6.0%; free-lance profession: 2.1%; part-time worker: 2.7%; student: 1.4%; housewife: 3.1%; not employed: 0.2%; other: 2.5%; N/A: 9.7%). Presumably because this was an alumni survey, and the location of the university was in *Hyogo*, the distribution of the analyzable graduates contained many participants from *Osaka* (JCS = −5.11) and *Hyogo* (−2.7) prefectures. Also, the residence of our sample was largely from the individualistic half of the country (Supplementary Material, Appendix 1); therefore, the results should not be understood to cover full range of *prefecture* variation in Japan. Despite this limitation, a wide age range from working to retired adults who had been working during 1990 to 2010 were obtained, which is valuable for the purpose of this study.

^17^Although Kijima et al. (1996) reported reliability and retest reliability using the TCI, items we used showed low reliability possibly due to small number of items we used. Future studies need to measure personality variables using more reliable measures.
