# Supplementary material for: Living in the tide of change: explaining Japanese subjective health from the socio-demographic change
Source: Front Psychol. 2014 Oct 29;5:1221. doi: 10.3389/fpsyg.2014.01221 (PMC4212602; doi:10.3389/fpsyg.2014.01221)
Supplement: Supplementary file 1 [file DataSheet1.DOCX]

Appendix 1

Frequency distribution of JCS.

*Note*. ^1^ JCS is a prefecture level score, with a higher positive indicate higher collectivistic/

lower individualistic socio-demographic condition of the prefecture.

^2^ Percentage of participants in the sample.

Appendix 2

Frequency distribution of JCSCL.

*Note*. ^1^ JCSCL is a city level score, with a higher positive indicate higher collectivistic/

lower individualistic socio-demographic condition of the city.

^2^ Percentage of participants in the sample.

Appendix 3

Intra-Class Correlations among Two Levels of Regional Differences and Health Measures (Explained Variables).
